# Guidelines for the ethical review of laboratory animal welfare People’s Republic of China National Standard GB/T 35892‐2018 [Issued 6 February 2018 Effective from 1 September 2018]

**DOI:** 10.1002/ame2.12111

**Published:** 2020-04-14

**Authors:** Judy A. MacArthur Clark, Deming Sun

**Affiliations:** ^1^ JMC Welfare International Sandwich, Kent UK; ^2^ National Research Institute for Health & Welfare and Ethics Committee of Chinese Association for Laboratory Animal Science Beijing China

## Abstract

These Chinese National Guidelines (GB/T 35892‐20181) were issued February 06, 2018 and became effective September 01, 2018. The authors recognized the urgent need for an authentic English translation to inform the international community of the compliance requirements in China. It was appreciated that the final translation must reflect the specialist understanding of those working under the Guideline whilst remaining faithful to the meaning of the original Chinese text. A three‐step translation process was therefore determined.

Step 1: A professional interpretation service (KL Communications, UK) was commissioned to prepare a literal translation of the Chinese text. Supportive documents were provided which explained specialist terminology. This translation was checked by two bilingual experts.

Step 2: A workshop was held in Nanjing in May 2019 to which were invited experts in laboratory animal welfare and ethical use. These included international native English‐speaking and Chinese‐speaking delegates. The delegates worked in multi‐lingual teams to review sections of the literal translation ahead of the workshop, and to agree an authentic interpretation during the workshop.

Step 3: Following the workshop, three bilingual experts (two native Chinese speakers and one native English speaker) reviewed the entire document to ensure consistency of terminology and general accuracy. This document is thus not a “literal translation” but an “accurate interpretation” of the original text. Any challenge of work being performed under these Guidelines should rely on the Chinese text in the first place. However, this translation may be used as mitigating evidence, especially where those performing the work are non‐Chinese speakers.

## CONTENTS

This document is the agreed English translation of the Guidelines for the ethical review of laboratory animal welfare^1^ prepared as described in the abstract. The original Chinese version was drafted in accordance with the criteria set out in Standard GB/T1.1‐2009.

**Table:** 

Foreword	
1.	Scope
2.	Standardization References
3.	Terms and Definitions
4.	Review Bodies
5.	Review Principles
6.	Review Details
6.1.	Qualifications of Personnel
6.2.	Research Facilities
6.3.	Laboratory Animal Veterinarians
6.4.	Animal Sourcing
6.5.	Standard Technical Procedures
6.6.	Animal Husbandry
6.7.	Animal Use
6.8.	Occupational Health and Safety
6.9.	Animal Transport
7.	Ethical Review Procedures
8.	Project Application Review Criteria
9.	Record Management

## FOREWORD

This document is drafted in accordance with the criteria set out in Standard GB/T1.1‐2009.

This document is initiated and overseen by the Technical Committee for Laboratory Animal Sciences of the Standardisation Administration of China (SAC/TC281).

Organizations involved in the drafting of these Guidelines include the National Research Institute for Health; National Institutes for Food and Drug Control; Beijing Cancer Hospital; Sanofi (China) Investment Co., Ltd.; Institute of Laboratory Animal Sciences Chinese Academy of Medical Sciences Laboratory Animal Centre, Peking University; the Welfare and Ethics Committee of the Chinese Association for Laboratory Animal Sciences (CALAS).

Major authors involved in the drafting of this document: Deming Sun, Bingfei Yue, Rongze Sun, Tianqi Wang, Wanyong Pang, Qi Kong, Desheng Zhu, Nan Li, Chuan Qin.

## 1 SCOPE

This Standard stipulates the requirements of ethical review and animal welfare management in the production, transportation and use of laboratory animals, and covers the establishment of the review body, review principles, review details, review procedures, review criteria, and record management.

This Standard is applicable to the ethical review and welfare of laboratory animals, and quality management thereof.

## 2 STANDARDIZATION REFERENCES

The following references are essential to the application of this document. For dated references, only the cited edition applies. For undated references, the latest edition of the document referred to (including any amendments) applies.

GB14922.1 Laboratory animal—Standards and monitoring for parasitology

GB14922.2 Laboratory animal—Microbiological standards and monitoring

GB14923 Laboratory animal—Genetic quality control of mammalian laboratory animals

GB/T14924.2 Laboratory animal—Hygienic standard for formula feeds

GB14924.3 Laboratory animal—Nutrients for formula feeds

GB14925 Laboratory animal—Requirements of environment and housing facilities

Guidance on the Care of Laboratory Animals: (2006) No. 398, Ministry of Science and Technology

## 3 TERMS AND DEFINITIONS

For the purpose of this document, the following terms and definitions apply.

### 3.1 Laboratory Animal

An animal used in research, teaching, breeding, testing, and other scientific experiments.

### 3.2 Laboratory Animal Welfare

The totality of the concept of ensuring the health, psychological well‐being, and the quality of life of laboratory animals, as well as the provisions made to achieve it.

### 3.3 Laboratory Animal Ethics

In both the treatment of laboratory animals and the conduct of animal experiments, compliance with the standards and principles of societal morality.

### 3.4 Environmental Enrichment

The optimization and enrichment of living conditions for laboratory animals (for instance, housing standards, cages, toys, level of comfort, foraging opportunities, ability to carry out natural behaviors etc.).

### 3.5 Ethical Review

In accordance with the principles and standards of laboratory animal welfare and ethics, an examination and assessment expressly conducted on the necessity, rationality and compliance with relevant regulations of the use of laboratory animals.

### 3.6 Humane Endpoint

An endpoint which is applied at the earliest possible stage when an animal is exhibiting pain or suffering during the course of the experiment, when it has become clear that the experimental objectives have been met, or that they cannot be met.

### 3.7 Euthanasia

A method of humanely ending the life of an animal causing peaceful and rapid death while, to the greatest extent possible, reducing or eliminating the animal’s fear and suffering.

### 3.8 The principles of the 3Rs

The principles of Replacement, Reduction and Refinement in the use of laboratory animals.

#### 3.8.1 Replacement

Methods which replace animals of a higher life form with those of a lower life form, or avoid the use of animals and employ alternative methods to achieve the same objectives as experiments which use animals.

#### 3.8.2 Reduction

Methods which minimize the number of animals used per experiment to obtain the necessary data that support the scientific objectives.

#### 3.8.3 Refinement

When laboratory animals must be used, the minimization as far as possible of the frequency of use of inhumane methods or of the degree of harm caused.

### 3.9 Care of laboratory animals

The effective care given to laboratory animals by humans to safeguard their welfare and interests and to avoid causing them unnecessary harm.

### 3.10 The five freedoms

Through the promotion of animal welfare, ensuring for animals the Five Freedoms or Five Rights to enjoy comfort, good health, and psychological well‐being, including:

- Freedom from thirst and hunger—by ensuring animals have access to fresh water and food to maintain their health and vitality.
- Freedom from discomfort—by providing animals a comfortable living environment.
- Freedom from pain, harm and disease—by putting in place preventative care, and diagnosing and treating animals in a timely manner.
- Freedom to carry out most natural behavior—by providing sufficient space, appropriate facilities, and company of the animal’s own kind.
- Freedom from fear and distress—by ensuring good conditions and handling that will not cause mental distress or suffering to the animals.

## 4 REVIEW BODIES

### 4.1 Establishment of the review body

4.1.1 According to particular areas of management authority, review bodies may be established at different administrative levels. The review body may belong to a local/regional Laboratory Animal Welfare and Ethical Review Administration or to an individual institution. Administrations or institutions are responsible at their respective levels for establishing their review bodies and appointing members.

4.1.2 The review body is a specialized organization that independently conducts reviews and assessments. It may be variously designated “Animal Welfare and Ethical Review Body (AWERB)”, “Institutional Animal Care and Use Committee (IACUC)” etc., but all such bodies shall have the function of review, and are hereinafter referred to as “Ethics Committees”.

### 4.2 Scope of responsibilities of the Ethics Committee

#### 4.2.1 General

The Ethics Committee is responsible for reviewing and overseeing the conduct of institutions within its jurisdiction on animal welfare and ethics, according to the provisions of laboratory animal related laws, regulations, and quality and technical standards. The Ethics Committee also deals with misconduct reports and complaints.

#### 4.2.2 Tasks

4.2.2.1 To inspect institutions’ management, operational policies, and processes every 6 months; to review and oversee animal use projects before, during, and after completion; to investigate any instances of non‐compliance with laws and regulations.

4.2.2.2 To independently carry out review and oversight; to issue review and inspection reports; to provide reports to the institute management and to the senior administrative body supervising the institute.

### 4.3 Membership of the Ethics Committee

4.3.1 The Ethics Committee shall comprise at least one of each of the following: laboratory animal specialist, veterinarian, laboratory animal management staff, researcher using laboratory animals, lay member of the public etc. No more than three members from the same department or branch of an institution or body may sit on the Committee at a time. The Committee will appoint one chair, and a number of vice‐chairs and Committee members as the review work practically requires.

4.3.2 Ethics Committee members are appointed for 3 to 5 years. The review body which establishes the Ethics Committee is responsible for appointments, induction and preliminary training, dismissals, and replacement of members in the case of vacancies.

4.3.3 All members of Ethics Committee must comply with the laws, regulations and standards, in order to uphold animal welfare and ethics.

### 4.4 Management of the Ethics Committee

The Ethics Committee shall establish a code of conduct for the Committee, along with systems and processes for reviewing, overseeing and holding regular meetings. It shall also produce a disciplinary code as well as a professional training plan. It is responsible for reporting to the senior administrative body with oversight of the Committee. Decision making in the Committee follows majority rule. However, comments and views expressed by the minority shall be properly recorded.

## 5 REVIEW PRINCIPLES

### 5.1 The principle of necessity

The husbandry and use of laboratory animals as well as any program of harmful experiments must be carried out based on thorough scientific significance and necessity. Purposeless over‐breeding, overuse, misuse, or unjustified killing of laboratory animals is prohibited. Duplication of experiments without purpose is prohibited.

### 5.2 The principle of protection

For projects that are deemed necessary the Principles of the 3Rs shall be followed to ensure humane protection of laboratory animals. Where the scientific validity of the results of a program of experiments is not compromised, as far as possible alternative [“Replacement”] methods should be adopted, use of unnecessary numbers of animals should be reduced, and the frequency and severity of harmful use of animals should be reduced.

### 5.3 The principle of welfare

As far as possible, the humane care of laboratory animals shall be assured. During the lifetime of a laboratory animal, including during transport, to the greatest possible extent it should enjoy the Five Freedoms, and its opportunity to experience a natural life, health and well‐being should be guaranteed. Management and treatment of any species of laboratory animal should comply with the operating procedures stipulated for that species. Unnecessary stress, suffering and harm should be prevented or reduced, and animals should be treated in a manner which causes the least suffering.

### 5.4 The principle of ethics

The lives and interests of animals shall be respected while at the same time the principles of human societal morality shall be observed. Should brutal or inhumane treatment toward animals occur, it shall be stopped. Experimental objectives, methods and procedures shall conform to moral and ethical values accepted by human society and to internationally recognized practice. Laboratory animal projects shall ensure the safety of practitioners and the public environment.

### 5.5 The principle of the balance of interests

The conclusions of a welfare and ethics review of a project involving animal experiments should be arrived at responsibly, after a process that pays regard to the moral and ethical values accepted by contemporary society, and takes into account the interests of animals and humans, comprehensively and objectively assessing harms suffered by animals and the possible benefits derived as a consequence by humans.

### 5.6 The principle of fairness

The review and supervision shall be independent, just, fair, scientific, democratic, and transparent; shall not breach confidentiality, and shall be free from interference by political, commercial or personal conflicts of interest.

### 5.7 The principle of legality

There shall be no violation of laws and regulations or non‐compliance with relevant standards with regard to project objectives, sources of animals, facilities, personnel qualifications, operating procedures, or any other aspect.

### 5.8 The principle of taking account of national constraints

The welfare and ethical review shall comply with internationally recognized codes and standards, whilst being in line with China’s traditional public order, moral values as well as prevailing constraints, and shall reject radical ideas and extreme practices of any kind.

## 6 REVIEW DETAILS

### 6.1 Qualifications of personnel

6.1.1 All people working in the animal program shall have undergone professional training and acquired the necessary qualifications and skills.

6.1.2 Institutions shall develop professional training plans for personnel, and execute the training plan on laboratory animal welfare and ethics in accordance with actual needs. Institutions shall ensure that the personnel are familiar with the regulations and standards of laboratory animal welfare and ethics; that the personnel have a good understanding and knowledge of laboratory animal care; that the personnel have obtained knowledge of the behaviors of the relevant species as well as correct operating procedures.

6.1.3 When a new person or trainee is introduced to the institution, and if that person is involved in experiments which use live animals, guidance and supervision shall be provided under an experienced professional until the newcomer is proficient in procedures using live animals.

6.1.4 During laboratory animal technical training and the teaching practice of welfare and ethics, if alternative non‐animal methods can effectively reduce or replace the use of living animals and still achieve the training objectives, teaching methods involving replacement of living animals shall be used as far as possible.

### 6.2 Research facilities

#### 6.2.1 Animal facilities

Facility conditions and environmental parameters for laboratory animal breeding and use shall meet the requirements of Standard GB14925 as well as the national relevant regulations on laboratory animal care. All animal facilities shall have obtained an administrative animal‐related permit.

#### 6.2.2 Facilities and equipment

The quality of laboratory animal housing equipment and bedding shall comply with Standard GB14925, laboratory animal welfare standards and relevant national management regulations on the care of laboratory animals. It shall be ensured that the facilities and equipment do not cause accidental injury or harm to animals.

#### 6.2.3 Use of equipment

Housing equipment shall be cleaned and disinfected regularly; bedding shall be sterilized, have minimal dust, and be replaced regularly, as well as kept clean and comfortable; drinking and feeding systems for animals shall be safe, reliable and easy to use, as well as kept clean, sanitized, and in good working condition.

#### 6.2.4 Housing space

The minimum floor‐area (space) and height of cages housing any species of animals shall comply with relevant laboratory animal standards. Space shall be allocated appropriate to the husbandry requirements and physiological and behavioral needs of the animals. It shall be ensured that each animal caged is able to freely express most natural behaviors, including turning around, standing upright, stretching limbs, lying down, walking, grooming, nesting etc.

#### 6.2.5 Breeding environment

For pregnant or parturient animals, at least 10% greater cage floor area than set out in relevant standards normal for the species should be provided, with an environment and conditions suitable for giving birth.

#### 6.2.6 Environmental enrichment

The housing environment of animals shall be enriched by the provision of suitable items that encourage activities or play, in order to promote psychological well‐being. These items, however, should not harm the health and safety of animals or humans or prevent the achievement of the scientific objectives.

#### 6.2.7 Activity/play area

Nonhuman primates, dogs, pigs and other large laboratory animals that are naturally physically active and are used for breeding or involved in long‐term projects shall additionally be provided with an activity area and be exercised regularly. Items for enrichment and play appropriate to the species shall be placed in the activity area to enrich the environment.

#### 6.2.8 Regular inspection

The Ethics Committee shall inspect the animal facility regularly. Site inspections shall be conducted at least once every 6 months. The inspection shall include the details of implementation of projects that involve laboratory animals; animal housing conditions; operation and safety of facilities; hygiene, disease control and prevention; condition of cages and other equipment; housing density; animal health; environmental enrichment; compliance with standards of experimental procedures and surgery; occupational health of the staff and bio‐safety; and implementation of laboratory animal welfare and ethics standards.

### 6.3 Laboratory animal veterinarians

#### 6.3.1 Qualifications and training

Laboratory animal veterinarians shall be graduates in a veterinary or animal medicine‐related major discipline and shall have obtained the appropriate qualifications and received the relevant training. The review, implementation, and inspection of laboratory animal projects shall be carried out with the participation of a laboratory animal veterinarian or other person specialized in the veterinary care of laboratory animals.

#### 6.3.2 Scope of responsibilities

6.3.2.1 The laboratory animal veterinarian is responsible for conducting daily inspections, supervising and providing advice on animal welfare and ethics in the institution and the facility.

6.3.2.2 The laboratory animal veterinarian is responsible for animal disease prevention. The veterinarian shall be familiar with the prevention and control technicalities of diseases for each species in the facility, and shall be responsible for vaccination of animals, as well as developing and producing disease control protocols and quarantine programs for microorganisms, parasites, and other diseases.

6.3.2.3 The laboratory animal veterinarian is responsible for animal disease monitoring. This includes regular monitoring for the presence of parasitic, bacterial, and viral epidemics or infections, as well as subclinical infections.

6.3.2.4 The laboratory animal veterinarian is responsible for timely diagnosis and treatment of diseases. The veterinarian has the authority to give appropriate treatment to or take containment measures for animals that have been assessed and diagnosed with illness or injury and to perform euthanasia.

6.3.2.5 The laboratory animal veterinarian is responsible for planning animal‐use programs of research. The scientists involved in the experiment shall consult with a laboratory animal veterinarian to agree on a study plan that will achieve the experimental objectives.

6.3.2.6 The laboratory animal veterinarian is responsible for the management and use of controlled drugs.

6.3.2.7 The laboratory animal veterinarian is responsible for conducting post‐mortem examinations and the production of post‐mortem reports. In the case of sudden disease or abnormal death, based on the result of the postmortem report, the veterinarian shall advise on appropriate containment measures.

6.3.2.8 The laboratory animal veterinarian is responsible for taking medical notes and managing medical records, and preparing specific care and treatment plans.

6.3.2.9 The laboratory animal veterinarian shall participate in the ethical review and implementation of laboratory animal projects. The veterinarian shall supervise, inspect and provide professional judgment on the implementation of laboratory animal welfare and ethics.

6.3.2.10 The laboratory animal veterinarian is responsible for advising on measures to prevent and control zoonoses, identifying the source of disease in order to minimize risks. The veterinarian shall undertake appropriate preventative measures in the facility, such as ensuring personnel wear personal protective equipment, executing appropriate disinfection procedures, or using isolation or segregation to prevent disease outbreaks when needed. This is to reduce the risk of the disease spreading and to ensure bio‐safety.

6.3.2.11 The laboratory animal veterinarian shall advise on quarantine programs when new animals arrive at the facilities and carry out the appropriate inspections. If any infection is found that has a legal requirement to be reported, the veterinarian must notify the relevant government authority in a timely manner.

6.3.2.12 The laboratory animal veterinarian is responsible for advising and overseeing surgery and post‐surgical care, and participating in the inspection and oversight of pre‐, peri‐, and post‐surgical procedures, as well as inspecting and managing surgical and post‐surgical complications.

6.3.2.13 The laboratory animal veterinarian is responsible for providing professional guidance on, or performing the administration of, anesthesia, analgesia, and euthanasia.

6.3.2.14 The laboratory animal veterinarian shall advise on humane endpoints. The veterinarian shall be consulted and negotiate with researchers during project planning and before animal experiments. During experiments, when it has become clear that the experimental objectives have been met or cannot be met, the veterinarian shall identify and implement humane endpoints in a timely manner. In accordance with the ethical review plan, the veterinarian shall, when necessary, have the authority to reduce pain and stop unnecessary harm by performing euthanasia or through other means.

### 6.4 Animal sourcing

6.4.1 It is prohibited to use animals from unidentified sources, stolen or captured stray animals, or endangered wild animals.

6.4.2 All animals shall be marked or tagged for the purposes of identifying individuals or groups. Animal identification shall use a reliable method that is the least harmful or causes the least suffering to the animals.

6.4.3 For projects which necessitate the use of wild animals, animals shall be obtained through legal means and captured using humane methods. The health, welfare and safety of both humans and the animals shall be taken into consideration.

6.4.4 Field research shall not disturb the natural habitat nor have a negative impact on the targeted or any untargeted species.

6.4.5 A welfare and ethical review is required to assess the possible disturbance caused to animals taken from the wild. To avoid potentially severe or fatal consequences to the animals, effective measures should be taken to minimize any suffering or distress in the course of capture, transport, sedation, anesthesia, identification, and sampling etc., including their cumulative effects.

6.4.6 Endangered species may only be obtained and used in exceptional circumstances, and in accordance with the law. The use of endangered species is only permitted when there is no scientific basis for the use of alternatives and results cannot be obtained using other species. The project may only be implemented after prior review and approval.

### 6.5 Standard technical procedures

Laboratory animal husbandry, facility management, experimental procedures including the determination of humane endpoints and euthanasia for each species, environmental controls, and execution of all laboratory animal experiments should comply with laboratory animal welfare and ethics quality standards, management regulations and Standard Operating Procedures (SOP), and should be provided to the Ethics Committee for inspection and supervision during implementation.

### 6.6 Animal husbandry

6.6.1 Personnel must not tease or abuse laboratory animals. Capture of animals shall be by appropriate methods, with sympathetic and gentle handling in order to avoid causing animals discomfort, fear, pain, or injury.

6.6.2 In the course of routine husbandry animals shall be regularly observed. If abnormalities are detected the cause shall be promptly investigated and the necessary actions taken to address and correct them.

6.6.3 Animal care personnel shall provide animals with sufficient feed and clean drinking water in accordance with their feeding habits and nutritional needs. Nutritional content shall comply with the requirements set out in Standard GB/T14924.2. The microbiological and physical‐chemical properties of feed shall comply with the requirements set out in GB/T14924.3.

6.6.4 Newly received animals require a period of acclimatization before they are used in order for them to reach a state of physiological and behavioral stabilization. The duration of the acclimatization period depends on the mode and duration of transport, age, species, source, and anticipated use of the animals. It also depends on the differences between the environments and husbandry conditions of the place of origin and destination.

6.6.5 Animal facilities shall be able to effectively separate and isolate sick animals from healthy animals.

6.6.6 The specific nutritional needs of animals shall be fully met during gestation, lactation, at different stages of experiments, and during postsurgical recovery periods.

6.6.7 When an experiment requires animals’ food and water intake to be restricted, full scientific justification and explanation of the necessity for fasting must be provided and submitted to the Ethics Committee for prior review and approval.

6.6.8 Laboratory animal veterinarians or personnel trained in laboratory animal veterinary care are required to monitor the parturition of dogs, pigs, and other large animals to prevent unforeseen accidents. Hand rearing, nursing, and other necessary measures shall be available to pups, piglets etc., that are not able to live independently immediately after birth.

6.6.9 For projects involving the breeding and use of genetically altered animals and spontaneous or induced mutant models in which the mutation is harmful to the animals, animals shall be monitored and special husbandry and welfare‐related needs associated with the model phenotype shall be provided.

6.6.10 Hygiene and disease control and prevention of the housing and experimental environments shall meet the requirements set out in relevant regulations and standards. There shall be proper disease control and prevention protocols and husbandry management in place at facilities to avoid cross‐infection between animals and between humans.

6.6.11 When habitually social animals are housed individually, there shall be a sound reason for doing so, and effective measures taken to reduce the individual animal’s loneliness or suffering.

### 6.7 Animal use

6.7.1 In accordance with scientific principles, research into and application of laboratory animal alternatives shall be actively pursued. Unnecessary duplication of animal experiments shall be avoided.

6.7.2 The number of animals used shall be reduced to a minimum. The entry of unauthorized personnel to places where experiments are conducted shall be avoided.

6.7.3 When surgery or dissection is carried out on live animals, including when neuromuscular blockers are used, effective anesthesia shall be performed. Survival surgeries shall be conducted using aseptic techniques. During postoperative recovery, analgesics, appropriate nursing care, and dietary adjustments shall be provided based on the animal’s condition.

6.7.4 Restraint of animals shall be carried out in a way that as far as possible reduces their discomfort, suffering, and stress responses. Restraint devices shall be of appropriate structure, size and durability, and be safe, hygienic and easy to use. Physical restraint shall be used to the minimal extent required in order to meet the experimental objectives.

6.7.5 Animals shall be killed using appropriate methods of euthanasia. No other animals should be present where killing is being carried out. Only after death is confirmed shall an animal’s cadaver be disposed of appropriately.

6.7.6 Provided the experimental results have been obtained, a “humane endpoint” shall be selected at the first opportunity, reducing as far as possible the time the animal experiences suffering. With the exception of rare instances when the experiment requires it, death (except by means of euthanasia) should not be used as a study endpoint.

6.7.7 Wild animals that have not been adversely affected by an experiment and are no longer needed, for example those used in a control group, may be returned to their original habitat in accordance with the law once they have undergone a scientific examination and assessment.

### 6.8 Occupational health and safety

6.8.1 The institution shall have comprehensive occupational health and safety management regulations and standard operating procedures. The institution shall provide appropriate occupational health and bio‐safety training and personal protective equipment for personnel.

6.8.2 Based on the main safety risks at the facility, risk assessment, risk review and an emergency plan shall be developed and practice drills conducted. Examples of risks include zoonoses, toxic and hazardous chemical agents and biopreparations, radioactive hazards, allergens, specific high‐risk experimental procedures, animal attacks, and resulting injuries etc.

6.8.3 In the event that a biohazard reaches a level that may cause harm, appropriate biological containment and exclusion measures shall be put in place to ensure the health of personnel and animals. Further measures shall be taken to prevent the spread of the biohazard and to ensure the safety of the public environment.

6.8.4 The review focuses on the technical approach to ensure personnel health and safety, the safety of animal experiments, and public health.

### 6.9 Animal transport

#### 6.9.1 Transport requirements

6.9.1.1 Personnel who transport animals shall have the required laboratory animal practitioner qualifications. Measures shall be taken to reduce animal transport as much as possible. During transport, measures shall be taken to reduce animal stress responses. Housing environments and conditions before and after transport shall be as consistent as possible. After transport, experimental procedures shall not be carried out immediately, but rather, animals shall be given sufficient time to acclimatize to their new surroundings.

#### 6.9.2 Transport conditions

6.9.2.1 The shipping institution and the receiving institution shall confer and negotiate in advance, to agree an animal transport plan which includes carrier details, means of transport, transit route, transit duration, weather conditions, shipping containers, and contingency plans, in order to ensure that, for the animals, shipping occurs in the shortest, safest, and most comfortable way possible.

6.9.2.2 Implementation of the animal transport plan shall be carried out under the supervision of a laboratory animal veterinarian or laboratory animal specialist.

6.9.2.3 If an animal transport plan may have a negative impact on animal welfare, a welfare and ethical review shall be carried out.

6.9.2.4 It is not appropriate for animals to be transported while they are ill, in post‐operative recovery, near parturition, or at other times when they are unfit to travel.

6.9.2.5 Transport shall also comply with the requirements on laboratory animal transport set out in the “Guidance on the Care of Laboratory Animals”.

6.9.2.6 Environmental conditions during transport shall comply with the relevant requirements in Standard GB14925. The environment shall be safe and non‐hazardous to animals and shall ensure that for the entire transport process animals are comfortable and can breathe freely. Transport cages shall be safe, reliable and sturdy so as to prevent animals from being injured or otherwise harmed, or from escaping, and also prevent other animals from entering. Incompatible animals should be separated to prevent them from harming each other.

6.9.2.7 If the duration of transport exceeds 6 hours, sufficient and appropriate food and water shall be supplied to animals. If the duration of transport exceeds 24 hours, bedding as well as rest breaks shall also be ensured.

#### 6.9.3 Import and export

6.9.3.1 Cross‐border animal transport shall be carried out only when transport conditions are appropriate to the animals’ physiological and behavioral needs and the animals are disease‐free. Effective steps shall be taken to ensure transport cages and packaging are physically contained, so as to eliminate the possibility of cross‐contamination between animals and the environment.

6.9.3.2 The transport plan and implementation of the plan shall comply with the relevant laws and regulations and welfare and ethics regulations of both China and the destination country or region.

#### 6.9.4 Loading, unloading, and arrival

6.9.4.1 Personnel responsible for loading and unloading animals shall have laboratory animal practitioner qualifications or understand the basic requirements of laboratory animal welfare. They shall be proficient and skilled in transport procedures in order to reduce stress in animals during transport.

6.9.4.2 Social animals that are transported in pairs or groups shall, on arrival at their destination, as far as possible be maintained in the same pairs or groups during the acclimatization period as they were when transported.

## 7 ETHICAL REVIEW PROCEDURES

### 7.1 Application materials

7.1.1 The person responsible for applying for the welfare and ethical review of the project shall formally submit the completed Application Form for Ethical Review and relevant supporting materials to the Ethics Committee. The application shall include the following:

- Title and overview of the project involving animal use;
- Name of the project leader and persons involved, including their professional training background, laboratory animal or animal experiment training certificate numbers, and license number of the animal facility;
- Project objectives, necessity and significance, design of the experiments, information on animals that the project intends to use (including species, quantity, and reason for selecting to use that species), anticipated level of pain and distress for the animals and preventive measures to take (e.g. anesthesia, analgesia, humane endpoints, and euthanasia, etc.), main steps taken for animal replacement, reduction, and refinement, and harm‐benefit analysis.
- Declaration of intention to comply with the principles of animal welfare and ethics.
- Any other information and documents requested by the Ethics Committee.

7.1.2 For a template of the Application Form, please refer to Appendix A of the original Chinese text (GB/T 35892‐2018)1.

### 7.2 Application review

7.2.1 After receiving the application, the Chair of the Ethics Committee designates experienced members to conduct a preliminary review.

7.2.2 In the case of a project which may be carried out routinely, after the initial application has undergone review by the Committee, the Chair or authorized vice‐Chair may sign off on subsequent reviews directly. All new projects shall be reviewed by the entire Ethics Committee which shall provide its views in written form within 5 working days. In case of any dispute, relevant external experts shall be consulted during a further Ethics Committee meeting to review the dispute.

7.2.3 Project application review shall involve more than half of the Committee members. The applicant may request to be present during the Committee meeting to answer any questions, and is entitled to make recusal requests for any Committee members who might have a conflict of interest in order to ensure confidentiality and impartial review of the project application.

7.2.4 The Committee shall reach unanimous decisions through discussion. If this is not possible, then the majority rule of the Committee carries, and welfare and ethical review of the application is decided within 10 working days. After sign‐off by the Committee Chair or vice‐Chair, the applicant will be notified of the final decision within 3 working days.

### 7.3 Post‐approval monitoring

7.3.1 The Committee shall monitor project activities through regular inspections to ensure the study is carried out in accordance with Committee‐approved animal use applications. The Committee shall suggest corrective actions whenever minor deficiencies are identified, as well as suspend any project if serious non‐compliance is found.

7.3.2 The project shall be carried out in accordance with the animal use application approved by the Committee. Any significant change or study modification involving laboratory animals which may have a negative effect on the welfare of the animals shall be reviewed and approved before the changes are implemented. The project plan shall specify the main responsibilities of the personnel involved.

7.3.3 Significant study modifications which may have a negative effect on the animals include:

- Experimental design, including change of species, quantity, source, as well as the rationality of selecting such species, including repeated use;
- Experimental procedures and technical methods;
- Transport and handling methods and special restrictions;
- Any increase in distress due to animal training, husbandry, restraint, or experimental manipulation;
- Methods to prevent or alleviate pain, discomfort, distress, or permanent disruption to the animals’ normal physical condition or physiological function, including the use of anesthesia, analgesia, and non‐pharmaceutical approaches to improve comfort level, such as provision of supportive care, supplemental heat source, softer bedding, assisted feeding, etc.
- The application of humane endpoints and final disposal of the animals, including euthanasia;
- Animal health, husbandry and care, including the provision of environmental enrichment;
- Study modifications which compromise the principles of “replacement, reduction and refinement” as well as the Five Freedoms;
- Any study components with health and safety hazards;
- Facilities, equipment, environmental conditions, and surgical procedures;
- Any change in key personnel in charge of the project and/or those responsible for conducting the experiments;
- The significance, objectives, scientific value, and societal benefit of using animals in the project (for example, harm‐benefit analysis);
- Other issues concerning the project that may negatively impact on the principles of laboratory animal welfare and ethics.

### 7.4 Review upon project completion

Upon project completion, the project leader shall submit a final report to the Ethics Committee on its conduct of welfare and ethics throughout the duration of the project, and undergo a retrospective review from the Ethics Committee.

## 8 PROJECT APPLICATION REVIEW CRITERIA

### 8.1 Application approval

If a project has not been found to be in breach of laws and regulations governing laboratory animal welfare and ethics, including these Standards, the Ethics Committee shall deem the project to have passed the welfare and ethics review, and produce a review report.

### 8.2 Application rejection

The Ethics Committee may reject an application under one of the following conditions:

- A project that refuses or avoids ethical review;
- A project that has not provided sufficient supporting evidence or materials, or has provided incomplete or false information;
- A project that lacks objective justification and necessity for its implementation or for causing harm to animals.
- A project which allows personnel without specialized training or relevant qualifications to be involved directly in animal breeding, transport, and use, or a project which is otherwise clearly non‐compliant with the principles and management requirements of laboratory animal welfare and ethics.
- A project where the work environment is unable to meet the relevant quality standards associated with animal breeding, transport, or experimentation; or where experimental conditions are unable to comply with animal welfare, occupational health and safety, public environmental safety requirements, or where there is failure to comply with animal feed, caging, and bedding standards.
- A project that lacks operating procedures for ensuring animal welfare and ethical conduct of personnel during animal breeding, transport, and use, or a project initiated without following standard operating procedures; a project that is found to be abusing animals, causing animals to experience undue stress, harm, disease, and death;
- A project whose design is flawed and unscientific or whose implementation does not reflect the Principles of the 3Rs, the Five Freedoms, or principles of animal welfare and ethics.
- A project whose design or implementation fails to reflect humane treatment of animals or show respect for animal life, or fails to refine or perfect experimental procedures in order to minimize animal pain and distress, or to reduce the number of unnecessary animal deaths, or to use the most effective euthanasia methods to reduce the duration and severity of animal suffering.
- A project that includes inappropriate use of anesthesia during surgery or prior to necropsy or fails to adhere to ethical and moral standards when handling live or dead animals, or uses extreme and controversial experimental procedures.
- A project that involves experimental methods or objectives that fail to adhere to national traditional moral and ethical standards, or fall outside internationally accepted norms, or which are expressly prohibited by the State, or violate current social expectations and scientific ethics.
- A project that causes animal harm without scientific merit or application nor practical benefit for humans or animals.
- A project which uses new scientific technology in animal experiments without moral or ethical restraints, violating traditional reproductive ethics, such as creating human‐animal hybrids by placing animal cells into human embryos or vice versa, or any experiments which undermine human dignity or which may cause huge ethical conflict in society.
- Any other actions that are in serious violation of relevant laboratory animal welfare and ethics laws, regulations, and provisions outlined in these guidelines.

### 8.3 Appeal and response to appeal

8.3.1 In cases where the result of the animal welfare and ethical review is disputed, the applicant or persons reviewed may resubmit the application providing new additional information or after making improvements to the original, or may make an appeal to the Ethics Committee at the next highest level.

8.3.2 Upon receipt of a resubmitted application or appeal, the Ethics Committee shall respond in writing within 10 working days.

## 9 RECORDS MANAGEMENT

9.1 The Ethics Committee shall designate a specific person in charge of receiving, sending, and managing documents, as well as document archiving.

9.2 All materials for review or inspection shall be filed and archived. A report of the reviews and inspections shall be signed by each Committee member who has participated in the review or inspection.

9.3 The review and inspection reports shall at least include the following: any violations of the laws and regulations related to animal welfare and ethics, and provisions contained in this Standard; deviations in implementation from the original approved project which compromise animal welfare; recommended corrective actions and the implementation of such actions; the opinions and the review conclusions from the Ethics Committee, and other critical information.

9.4 All documents of the Ethics Committee shall be filed and kept for not less than 3 years after the completion of the project.
